# Negative pressure pulmonary edema after laparoscopic cholecystectomy: A case report and literature review

**DOI:** 10.1097/MD.0000000000037443

**Published:** 2024-03-15

**Authors:** Xu Deng, Chun-Yuan Yang, Zong-Long Zhu, Wei Tian, Jian-Xing Tian, Ming Xia, Wei Pan

**Affiliations:** a Department of Hepatobiliary and Pancreatic Surgery, the People’s Hospital of Lezhi, Lezhi, China.

**Keywords:** extubation, general anesthesia, negative pressure pulmonary edema, pulmonary edema

## Abstract

**Rationale:**

Negative pressure pulmonary edema (NPPE) is an acute onset of non-cardiogenic interstitial pulmonary edema, commonly seen among surgical patients after extubation from general aneasthesia. It is mainly caused by rapid inspiration with acute upper airway obstruction resulting in significant negative thoracic pressure.

**Patient concerns:**

A 24-year-old female patient who underwent laparoscopic cholecystectomy under general anesthesia and developed NPPE postoperatively.

**Diagnoses:**

Her main clinical manifestation was coughing up pink foamy sputum; postoperative CT showed increased texture in both lungs and bilateral ground glass opacities.

**Interventions:**

Diuretics and steroids were used, and symptomatic supportive treatments such as oxygen were given.

**Outcomes:**

After treatment, on the fourth post-operative day, her symptoms were relieved and her vital signs were stable enough for her to be discharged.

**Lessons:**

Although this is a rare and severe complication, the prognosis of NPPE is good when it is managed with proper diagnosis and treatment.

## 1. Introduction

Negative pressure pulmonary edema (NPPE) is an acute onset of non-cardiogenic interstitial pulmonary edema.^[1–3]^ This is a very rare complication in patients after general anesthesia. According to previous case reports, NPPE often occurs in young, healthy patients.^[2,4,5]^ In this review, we report on a 24-year-old female patient who underwent laparoscopic cholecystectomy under general anesthesia and developed NPPE postoperatively. So, we describe the patient clinical symptoms and associated treatment measures.

## 2. Case report

The patient, a 24-year-old Chinese female, started to suffer from right upper abdominal pain after eating 6 months ago, with many recurrent episodes, which was treated with Ursodeoxycholic Acid Capsules to no avail. After several ultrasounds, which indicated that the gallbladder stones were increasing in size, she went to the hospital for a cholecystectomy. She had no clinical symptoms other than right upper abdominal pain, no history of “hypertension,” “congenital heart disease,” or other diseases, and no history of previous surgery.

### 2.1. Physical examination

She is a well-developed young woman with no disease other than gallbladder stones. She was 155 cm tall and weighed 49.5 kg with a BMI of 20.60 kg/m^2^. On admission, vital signs were stable, Temperature: 36.5°C; Pulse: 68 beats/minutes; Respiration: 17 breaths/minutes; Blood pressure: 110/68 mm Hg. On palpation, there was mild deep-pressure pain in the right upper abdomen, without rebound tenderness or muscle tension.

### 2.2. Diagnostic testing

Six months before surgery, the ultrasound showed gallbladder stones testing about 0.4 cm; 3 months later, about 0.7 cm, and before admission, about 1 cm. Preoperative cardiac ultrasound and chest X-ray were completed and showed no significant abnormalities; laboratory tests were not found to be substantial (Table 1).

**Table 1:** Relevant laboratory indicators before and after the onset of NPPE.

	Before NPPE	After NPPE
WBC (10^9^/L)	9.53	13.19
RBC (10^12^/L)	5.27	4.71
HB (g/L)	159	143
PLT (10^9^/L)	163	145
PT (s)	13.40	14.00
APTT (s)	24.40	26.00
D-Dimer (mg/L)	0.332	1.302
ALT (U/L)	38	27
AST (U/L)	30	22
ALB (g/L)	55.40	39.7
TB (µmol/L)	17.20	17.80
DB (µmol/L)	5.50	5.30
Urea (µmol/L)	4.18	1.67
Crea (µmol/L)	49.0	39.2
BNP (pg/mL)	-	103.8

NPPE = negative pressure pulmonary edema.

### 2.3. Hospital course

According to the standards of the American Society of Anesthesiologists (ASA), she had an ASA I. Dexmedetomidine was continuously pumped at 0.25 µg/kg/h, Midazolam 2 mg, sufentanil 15 µg, propofol 120 mg, and vecuronium bromide 5 mg were administered successively. After tracheal intubation, 2% sevoflurane was continuously inhaled, and remifentanil was continuously pumped at 0.05 µg/kg/min. An additional intraoperative dose of sufentanil 5 µg was administered, followed by neostigmine 1 mg, flumazenil 0.5 mg, and atropine 0.5 mg at the end of the procedure. When the patient was conscious, the endotracheal tube was removed. The entire procedure lasted approximately 1 hour, the anesthesia lasted 71 minutes, the intraoperative fluid volume was 400 mL, and the bleeding volume was about 5 mL. The urine volume was not recorded because a urinary catheter was not placed in place before the operation. During the procedure, all the vital signs were stable, and no abnormalities were observed.

She was admitted to the post-anesthesia care unit for 30 minutes and was transferred to the general ward as she did not show any clinical symptoms during the observation. Twenty minutes later, the patient began to cough pink frothy sputum, while diffuse wet rales could be heard on auscultation of both lungs. The patient further developed sinus tachycardia with a pulse range of between 122 beats/minutes and 130 beats/minutes. SpO_2_ dropped to 80% to 90% with the nasal cannula with oxygen flow at 3 L/minutes, respiratory rate was 20 to 25 breaths/minutes, and no symptoms such as respiratory distress were observed. Blood pressure was within the normal range of 100 to 115/70 to 75 mm Hg. An arterial blood gas analysis was immediately completed and revealed PaCO_2_ of 45 mm Hg, PaO_2_ of 61 mm Hg, and SaO_2_ of 90% (Table 2). The possibility of pulmonary edema was considered. After consultation with the cardiology, respiratory and anesthesiology departments, we initially ruled out the diagnosis of cardiogenic pulmonary edema and considered the diagnosis of NPPE because of the patient previous good physical condition and the approximately normal preoperative laboratory indices and imaging findings. Treatment was started with a 20 mg furosemide diuretic, and a urinary catheter was placed to record urine output, followed by 3 days of treatment with methylprednisolone sodium succinate 80 mg to prevent progression of the disease. Because the patient oxygen saturation could be maintained at about 90, we only administered mask oxygen without tracheal intubation and ventilator-assisted therapy.

**Table 2:** Arterial blood gas (ABG) results after the onset of NPPE.

	ABG taken after 30 min	ABG taken after 18 h
PH	7.35	7.39
PaCO_2_ (mm Hg)	45	40
PaO_2_ (mm Hg)	61	171
HCO_3_^−^ (mmol/L)	23.9	24.5
SaO_2_ (%)	90	100
Lac	1.1	0.9
FiO_2_ (%)	37.0	33.0

NPPE = negative pressure pulmonary edema.

**Figure 1.**
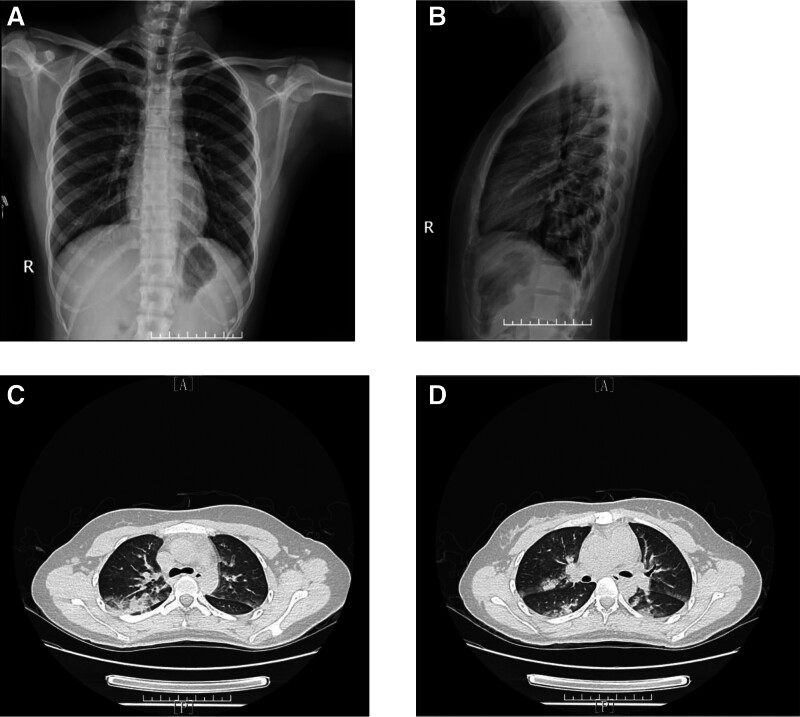
(A and B) Patient preoperative chest radiograph, no significant abnormality; (C and D) patient chest CT after NPPE, massive patchy hyperdense shadow in both lungs. NPPE = negative pressure pulmonary edema.

With the above treatment, her cough improved on the first day after surgery and her sputum was white mucus sputum instead of pink foamy sputum. A chest CT was also completed on the morning of the first postoperative day, showing increased texture in both lungs and bilateral ground glass opacities (Fig. 1). And some important laboratory parameters were also repeated and no relevant clinically significant abnormalities were found (Table 1). Due to the relief of her cough symptoms, diuretics were discontinued on the second postoperative day, but treatment with methylprednisolone was continued and maintained until the second postoperative day in order to continue to reduce respiratory edema and anti-inflammatory therapy. After discontinuation of the medication, the patient vital signs were stable and she was therefore discharged on the fourth postoperative day. At follow-up, no further relevant chest radiographs or CTs were done because the patient was preparing for pregnancy.

## 3. Discussion

NPPE is a rare and severe but completely curable form of non-cardiogenic pulmonary edema. Among patients undergoing surgical procedures, it is commonly seen as an acute upper airway obstruction following extubation under general anesthesia. The radiological manifestation of NPPE due to upper airway obstruction was first reported in 1973.^[6]^ According to studies, the factors that trigger the development of NPPE can be grouped into 2 categories: one is acute upper airway obstructive factors, including contractures of airway smooth muscle (e.g., laryngospasm), compression (tumor compression, inflammatory edema compression, tissue compression, and foreign body compression); and the other is caused by chronic obstructive diseases, mostly seen in sleep apnea syndrome, tonsillar and adenoidal hypertrophy.^[2,7,8]^ The incidence of postoperative laryngospasm has been reported to be 0.01% to 0.1% of all anesthetic procedures and is the most common cause of NPPE in adults, accounting for about 50% of reported cases.^[8–11]^

The most important factor causing NPPE is the rapid generation of a large negative pressure in the thoracic cavity during suctioning (Müller maneuver) of the obstructed voice box, which causes significant negative thoracic pressure.^[12,13]^ The main manifestation is that some patients take one or several deep breaths violently during general anesthesia awakening, and the laryngeal edema caused by tracheal intubation is blocked, leading to acute upper airway obstruction. The normal human chest pressure is −2 to −5 cmH_2_O, but in cases of severe acute upper airway obstruction, the pressure can reach −100 cmH_2_O.^[8]^ Inspiratory airway obstruction and vigorous respiratory motion are thought to be the 2 main factors contributing to NPPE. The main determinants of the rate of pulmonary edema formation are the transvascular hydrostatic pressure, the protein osmolarity gradient, and vascular permeability. When the rate of interstitial fluid accumulation exceeds the capacity of lymphatic drainage, pulmonary edema may develop, as evidenced by decreased arterial oxygen saturation, coughing up pink foamy sputum, or the appearance of new opacities on chest radiographs.^[8,12]^ It has also been suggested that hypoxia and increased capillary hydrostatic pressure might lead to rupture of alveolar capillaries and disruption of barrier function, leading to an increase in their permeability and contributing to the formation of pulmonary edema.^[13]^

According to studies, common risk factors for the development of NEEP after surgery included younger patients, males, ASA grade I to II, difficult intubation, head and neck surgery, obesity, and patients with history of recent respiratory infections.^[2,4,5]^ In adults, young and healthy males were more likely to produce greater inspiratory force, which could induce extremely high negative intrathoracic pressure when upper airway obstruction developed due to laryngospasm can be explained by “post-extraction vocal occlusion.”^[2,14]^ Meanwhile, elderly patients were often unable to generate sufficient negative chest pressure to develop pulmonary edema.^[8]^ In the pediatric population, the most common cause of obstruction is subglottic obstruction due to acute infectious laryngitis or epiglottitis.^[12]^

In most reported cases, NEEP usually occurs quickly, with only a few minutes between the onset of airway obstruction and the onset of pulmonary edema symptoms.^[13]^ The most common clinical presentation of patients with NPPE is frothy pink sputum from the mouth or tracheal tube and wet rales on auscultation of both lungs. Severe cases may present with progressive dyspnea and cyanosis, and hypoxia. A chest X-ray usually shows bilateral alveolar infiltrates or interstitial opacification, while CT mainly shows bilateral ground glass opacities.^[8]^ In this case, however, the patient first presented with coughing pink foamy sputum about 20 minutes after being taken out of the post-anesthesia care unit. It was the patient frequent coughing up of this sputum that drew our attention, and after consultation with the relevant departments, we began targeted treatment.

If properly treated, most patients with NPPE will have a resolution of pulmonary edema signs within 12 to 48 hours,^[14]^ with a small number resolving within 4 hours.^[15]^ Relief of expiratory obstruction is necessary, probably because when the acute upper airway obstruction is resolved, the negative pressure in the pleural cavity begins to decrease, exudation continues to decrease, some of the accumulated fluid is coughed up as sputum, and some is reabsorbed via lymphatic reflux, gradually reducing the signs of pulmonary edema.^[14]^ Low-tidal-volume ventilation may be beneficial. Although the area which has not been deeply studied was about ventilation for NPPE patients, a meta-analysis found a beneficial effect of low tidal volume ventilation on both the development of lung injury and mortality.^[12,16]^ However, in this case, the airway tended to become obstructed due to the Mueller maneuver. Hence, as the patient developed spontaneous breathing, the obstruction improved, and there was no need for reintubation with assisted mechanical ventilation as is the case in most NPPE patients.

In addition, the use of diuretics and steroids remains controversial, but both are commonly used in the treatment of patients with pulmonary edema. Some believe that NPPE is non-cardiogenic pulmonary edema and that the use of diuretics is of little use.^[14]^ Others recommend the use of small doses of diuretics. This is because they believe that it reduces blood volume and lowers pulmonary capillary pressure in the presence of normal renal function.^[12,17]^ Therefore, we used diuretics in the course of treatment. Although the use of steroids is controversial, we used it during treatment. This is because steroids have the following effects: reduce the inflammatory response and decrease capillary permeability; improve ventilation by relieving bronchospasm and decreasing intra-alveolar pressure; and promote diuresis by increasing renal blood flow and decreasing secretion of aldosterone and diuretic hormone. Therefore, in the treatment of this patient, we used methylprednisolone. Also, some researchers have suggested the addition of β agonists. Although NPPE may not be caused by bronchospasm, studies have shown that it can promote fluid clearance from the alveoli, thereby relieving the symptoms of pulmonary edema.^[12,14]^ Whereas in this patient, we did not use it. This was because the patient symptoms gradually started to improve after the use of diuretics and steroids and oxygen therapy.

## 4. Conclusion

NPPE is an acute onset of non-cardiogenic interstitial pulmonary edema. Its treatment remains an empirical one, as there is no consensus on its treatment and it is a rare postoperative complication of general anesthesia. We described our treatment course and provide one of our treatment strategies: in the presence of stable vital signs, oxygen can be administered by mask or nasal cannula (without the use of mechanical ventilation), combined with diuretics and steroid medication.

## Acknowledgments

The authors gratefully acknowledge all participants, especially the physicians and medical staff of cardiovascular medicine, respiratory medicine, and anesthesiology who helped us to make the correct diagnosis.

## Author contributions

**Conceptualization:** Xu Deng, Chun-Yuan Yang, Zong-Long Zhu, Wei Tian, Jian-Xing Tian, Ming Xia, Wei Pan

**Data curation:** Xu Deng

**Formal analysis:** Xu Deng, Wei Pan

**Investigation:** Xu Deng, Wei Pan

**Methodology:** Xu Deng

**Project administration:** Xu Deng

**Resources:** Xu Deng

**Software:** Xu Deng

**Supervision:** Xu Deng

**Validation:** Xu Deng

**Visualization:** Xu Deng

**Writing – original draft:** Xu Deng, Chun-Yuan Yang, Wei Pan

**Writing – review & editing:** Xu Deng, Chun-Yuan Yang, Wei Pan

## References

[1] Lemyze M Mallat J. Understanding negative pressure pulmonary edema. Intensive Care Med. 2014;40:1140–3. 24797685 10.1007/s00134-014-3307-7 PMC4148265

[2] Tsai P-H Wang J-H Huang S-C. Characterizing post-extubation negative pressure pulmonary edema in the operating room-a retrospective matched case-control study. Perioper Med (Lond). 2018;7:28. 30534363 10.1186/s13741-018-0107-6 PMC6282297

[3] Ghofaily LA Simmons C Chen L. Negative pressure pulmonary edema after laryngospasm: a revisit with a case report. J Anesth Clin Res. 2013;3:252. 24524005 10.4172/2155-6148.1000252 PMC3919040

[4] McConkey PP. Postobstructive pulmonary oedema--a case series and review. Anaesth Intensive Care. 2000;28:72–6. 10701042 10.1177/0310057X0002800114

[5] Bhaskar B Fraser JF. Negative pressure pulmonary edema revisited: pathophysiology and review of management. Saudi J Anaesth. 2011;5:308–13. 21957413 10.4103/1658-354X.84108 PMC3168351

[6] Capitanio MA Kirkpatrick JA. Obstructions of the upper airway in children as reflected on the chest radiograph. Radiology. 1973;107:159–61. 4266024 10.1148/107.1.159

[7] Benson A Schwarz M. A 26-year-old woman with recurrent hemoptysis and a sleep disturbance. Chest. 2008;134:1325–31. 19059964 10.1378/chest.08-1410

[8] Contou D Voiriot G Djibré M. Clinical features of patients with diffuse alveolar hemorrhage due to negative-pressure pulmonary edema. Lung. 2017;195:477–87. 28455784 10.1007/s00408-017-0011-8

[9] Goli AK Goli SA Byrd RP. Spontaneous negative pressure changes: an unusual cause of noncardiogenic pulmonary edema. J Ky Med Assoc. 2003;101:317–20. 14502951

[10] Pathak V Rendon ISH Ciubotaru RL. Recurrent negative pressure pulmonary edema. Clin Med Res. 2011;9:88–91. 20852091 10.3121/cmr.2010.936 PMC3134440

[11] Tebay A Bouti K Tebay N. Negative pressure pulmonary edema following a cholecystectomy—a case report. Rev Pneumol Clin. 2017;73:267–71. 29054715 10.1016/j.pneumo.2017.08.006

[12] Bhattacharya M Kallet RH Ware LB. Negative-pressure pulmonary edema. Chest. 2016;150:927–33. 27063348 10.1016/j.chest.2016.03.043

[13] Liu R Wang J Zhao G. Negative pressure pulmonary edema after general anesthesia: a case report and literature review. Medicine (Baltim). 2019;98:e15389. 10.1097/MD.0000000000015389 PMC6831334 31027133

[14] Krodel DJ Bittner EA Abdulnour R. Case scenario: acute postoperative negative pressure pulmonary edema. Anesthesiology. 2010;113:200–7. 20526178 10.1097/ALN.0b013e3181e32e68

[15] Budhathoki A Wu Y. Negative pressure pulmonary edema: a case report. JNMA J Nepal Med Assoc. 2020;58:491–3. 32827011 10.31729/jnma.4970 PMC7580407

[16] Serpa Neto A Cardoso SO Manetta JA. Association between use of lung-protective ventilation with lower tidal volumes and clinical outcomes among patients without acute respiratory distress syndrome: a meta-analysis. JAMA. 2012;308:1651–9. 23093163 10.1001/jama.2012.13730

[17] Wiedemann HP Wheeler AP Bernard GR. Comparison of two fluid-management strategies in acute lung injury. N Engl J Med. 2006;354:2564–75. 16714767 10.1056/NEJMoa062200

